# Applying methods of the global burden of diseases, injuries, and risk factors study to developmental neurotoxicants: a commentary

**DOI:** 10.1186/s12940-018-0397-7

**Published:** 2018-06-04

**Authors:** David C. Bellinger

**Affiliations:** Boston Children’s Hospital, 300 Longwood Avenue, Boston, MA 02115 USA

**Keywords:** Burden of disease, Developmental neurotoxicity, Subclinical toxicity, Disability weighting, Health loss

## Abstract

The purpose of this commentary is to consider whether the methods of the Global Burden of Diseases, Injuries, and Risk Factors Study (GBD) can provide accurate estimates of the impact of developmental neurotoxicant exposures on population health. The discussion focuses on two concerns. First, GBD implicitly largely endorses a “high risk” or “disease” approach to estimating health loss rather than a “population-based” approach. Exposure to many developmental neurotoxicants is highly prevalent but, for most individuals, it does not affect functional health to such an extent that diagnostic criteria for a disease are met. Nevertheless, the impacts are real and can be substantial when viewed in terms of the aggregate impact on a population. Second, in GBD the disability weights used for the most common sequelae of developmental neurotoxicant exposures, based on judgments provided by general population respondents, are not commensurate with the import that these sequelae have for an individual’s lifelong well-being, including their ability to fulfill educational, occupational, and social potential. It would be unfortunate if priorities were set or policy decisions made based on how developmental neurotoxicants compare to other risk factors using the current GBD methods.

## Background

The data generated over nearly three decades by the Global Burden of Diseases, Injuries, and Risk Factors Study (GBD) have been revolutionary in that a common metric, the Disability-Adjusted Life Year (DALY), is used to make direct comparisons within a population of the premature mortality and functional health losses attributable to a wide array of diseases and risk factors (e.g., [1]). GBD studies are thus intended to provide public health authorities with data that can be used to prioritize those interventions that will provide the greatest returns, in terms of health gains, on the investment of limited resources.

For any method that can be applied as broadly as that of the GBD, there are likely to be contexts in which some of the key assumptions are not fully appropriate. The purpose of this commentary is to examine whether such the GBD approach to characterizing “health loss” provides an accurate characterization of the impact of developmental neurotoxicants on population health.

## Main text

### Subclinical neurotoxicity

**Fig. 1:**
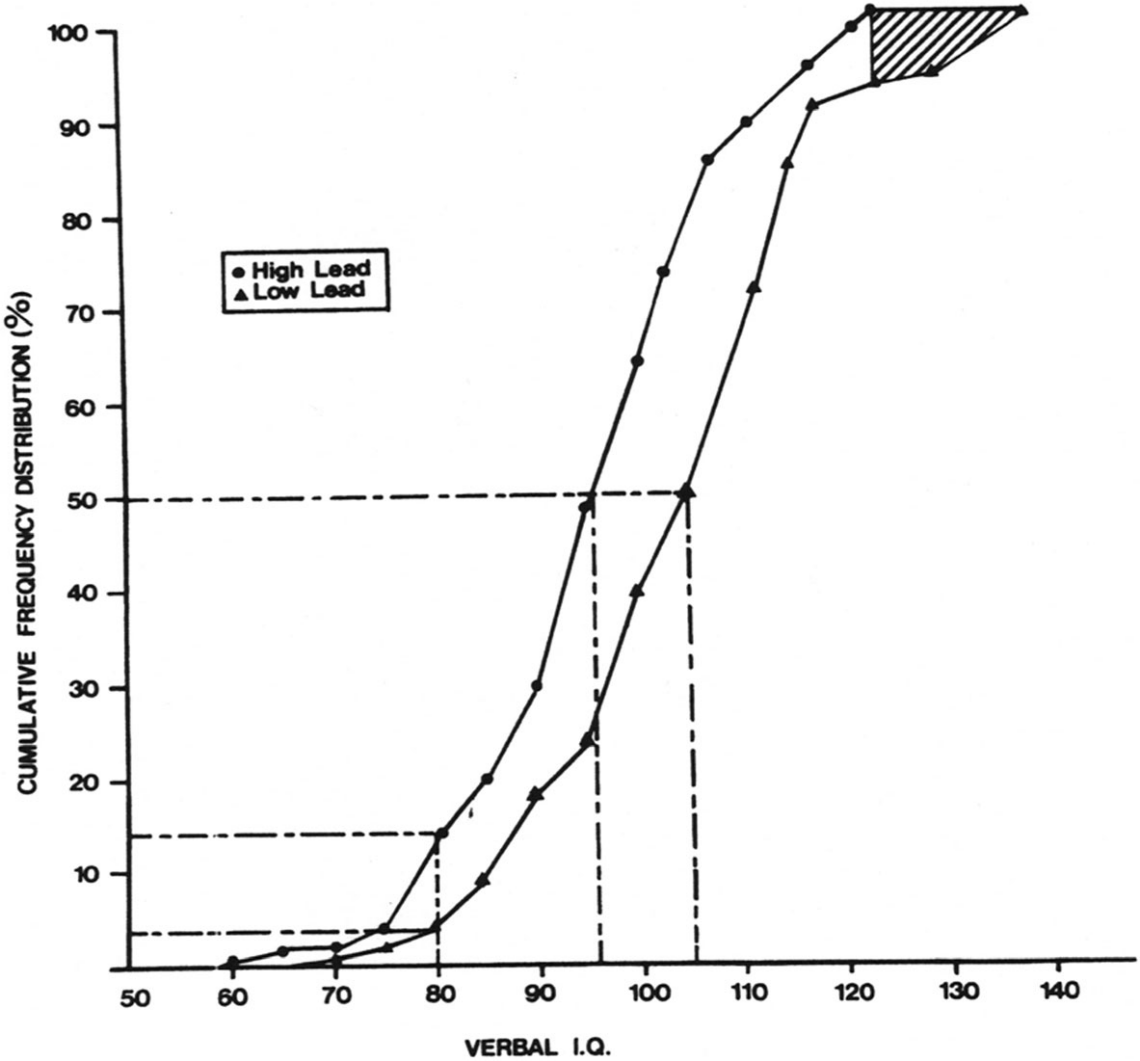
Verbal IQ Scores and Dentine Lead Concentration. Cumulative frequency distributions of Verbal IQ scores of children with a “low” (≤ 6 μg/g) or “high” (> 24 μg/g) concentration of lead in the dentine layer of deciduous teeth (from “Lead-associated intellectual deficit”, Needleman HL, Leviton A, Bellinger D, Volume 306, page 367. Copyright © 1983) Massachusetts Medical Society. Reprinted with permission

To date, relatively few studies have estimated the burden of disease associated with environmental chemical exposures. In analyses of lead [2] and methylmercury [3], the only health loss considered to contribute to the DALY total, at least with regard to developmental neurotoxicity, was an exposure-related reduction in IQ, specifically to a value below 70 (two standard deviations below the expected population mean). In the ICD framework, this is the criterion for identifying an individual with an “intellectual disability.” In the GBD 2015 analysis, in which lead was the only neurodevelopmental toxicant considered, “borderline intellectual disability” was also included, corresponding roughly to IQ scores of 70–84. It is certainly true that exposure to a neurotoxicant can cause the IQ scores of some children to fall below 70, but this would be expected to occur in a relatively small percentage of the exposed population, either individuals with extremely high exposures that cause frank brain pathology or individuals who, because of the presence of other significant risk factors, had an IQ score only a few points above 70 prior to the neurotoxicant exposure. Nevertheless, exposure-related changes in the numbers of individuals who fall into the lower portion of a performance distribution have frequently been used to illustrate the impact of a neurotoxicant on a population. For example, Needleman et al. [4] showed that the frequency of a Verbal IQ score below 80 was increased three-fold among children with “a high” versus “low” concentration of lead in the dentine layer of shed deciduous teeth (Fig. 1).

A fundamental problem with defining a health loss as occurring only when an individual’s IQ score falls below a certain value is that it lacks a logical foundation. There is no coherent rationale for considering a reduction in IQ from 73 to 68 be a health loss but not a reduction from 76 to 71. Use of cut-offs is necessary for a number of purposes, including to prioritize the allocation of treatment resources, third-party reimbursement, etc., but “health loss” is not a binary state. For many diseases, the relevant question is not whether an individual “has it,” but rather, “how much of it.” Fig. 1 also shows that the cumulative frequency distributions of the high and low dentine lead groups never crossed, only meeting when they finally converged at 100%. This leftward shift of the entire distribution of IQ scores, i.e., toward lower values, means that the exposure-associated reduction in IQ did not occur only among children functioning at the lower end of the distribution, but also occurred among children throughout the distribution. A complete reckoning of the burden attributable to lead must consider not only how the two distributions differ in their extreme tails but also the total area of the differences between the distributions. The exposure-associated reductions in the IQ scores of the children in the middle of the distribution, where vast majority of children fall, are, in aggregate, likely to contribute more to the total burden attributable to lead exposure than do the exposure-associated reductions in the relatively small number of children in the extreme lower tail of the IQ distribution. Bellinger [5] illustrated this by quantifying the impact, at the population level, of this leftward shift of the distribution, calculating the total number of IQ points lost from lead exposure in the cohort of U.S. children less than 5 years of age. When data on the dose-effect relationship, derived from a set of pooled analyses of prospective studies [6], were combined with NHANES data on the distribution of population blood lead concentrations, the total loss of IQ in this cohort was nearly 23 million points. This was substantially greater than the losses estimated for many other pediatric diseases and events, including ADHD (17 million points), traumatic brain injury (5 million points), congenital heart disease (100,000 points) and brain tumors (35,000 points). Because of the ubiquity of lead exposure and the absence of a threshold for neurotoxicity, nearly half of the total loss was contributed by the large number of children with a blood lead concentration less than 2.1 μg/dL. This is a variation on the principle, articulated by Rose [7], that, “a large number of people at a small risk may give rise to more cases of disease than the small number who are at high risk” (p.37). It is the cumulative effect of modest but prevalent impacts that are of greatest concern from the standpoint of population health. Developing a method for capturing the burden imposed by high prevalence-low morbidity exposures is crucial if the total morbidity associated with developmental neurotoxicant exposures is to be estimated accurately.

A calculation of the total IQ loss in the cohort of 0 to 5-year-old children from the late 1970’s suggests that the public health interventions implemented since the late 1970’s saved approximately 100 million IQ points insofar as the IQ loss attributable to lead in this earlier cohort of 0 to 5 year olds was approximately 125 million points. Given that the number of children in this age group is approximately 25 million, this represents an average increase of about 5 IQ points per child over this period, i.e., one-third of a standard deviation. Independent analyses suggest that the mean IQ score of U.S. adults has increased by 4 to 5 points over this same period.

Justification for considering a reduction in IQ as a health loss no matter where it occurs in the distribution is provided by the methods used in econometric analyses, in which each IQ point lost results in a reduction in lifetime earnings. For instance, in an analysis conducted using EU data, each IQ point lost was assumed to reduce lifetime earnings by 17,363 Euros [8].

Burden of disease analyses for neurotoxic chemicals must incorporate several considerations that might be less relevant to other risk factors. Although in econometric analyses the productivity loss per IQ point is assumed to be the same across the entire IQ distribution (i.e., the reduction an individual’s productivity is the same whether IQ drops from 120 to 119 or 75 to 74), this might be incorrect. In terms of import for future well-being, the loss of one IQ point for an individual whose pre-exposure IQ was low because of the presence of other risk factors might result in a greater reduction of future productivity than would the loss of one point for an individual whose pre-exposure IQ was higher. Also, there is evidence that individuals who are already low performers suffer disproportionately from a given increase in exposure to lead. Therefore, the dose-effect relationship assumed in estimating the impact of IQ loss should take into account the loss of function per unit increase in neurotoxicant biomarker at the relevant functional level of the individual.

The context-dependence of the severity of neurotoxicity also has implications for the estimation of disease burden globally. The distribution of co-morbid factors that modify neurotoxicity differs by region, so a given level of exposure might have more dire consequences for individuals in low-resource regions where the prevalence of some such factors might be greater than in high-resource regions. Thus, estimation of disease burden might require the use of region-specific assumptions about the magnitudes of the expected impacts. In addition, some environmental chemicals disrupt endocrine processes that affect brain development. Therefore, the possibility of sex-specific differences in the severity of neurotoxicity should be considered in estimating burden.

### Disability weights

A critical step in estimating the import of a nonfatal health state for the burden of disease is the assignment of a disability weight (DW). A DW can vary from 0 to 1, where 0 represents optimal health and 1 is equivalent to death. The DW and the duration of the imperfect health state, in combination, determine the Years-Lived-with-Disability component of the DALY total.

In the initial GBD studies, DWs were derived using economic evaluation methods. Health professionals provided judgments about the severity distribution of health states and the social preference for time lived at different severity levels. In effect, these were judgments about the import of the health state for quality of life, social desirability, and the value of the life of an affected individual. Responding to critiques of this approach, from GBD 2010 onwards the judgments about the value of departures from ideal health have been provided by individuals from the general population instead of health professionals. The method currently used involves the presentation of paired descriptions, in lay terms using 30 words or fewer, of individuals in two health states. The respondent is asked to decide, “who is healthier.”

The DWs assigned to certain health states linked to developmental neurotoxicant exposures appear to be greatly affected the training or experience of respondents. Table 1 lists the DWs for intellectual disability (ID) of different levels of severity used in GBD studies between 2004 and 2015 [9]. Health professionals, the respondents in GBD 2004, clearly regarded intellectual disability of all severity levels to entail a greater health loss than did members of the general population respondents in GBD 2010 and GBD 2015.

**Table 1:** Changes over time in disability weights for severity levels of intellectual disability in GBD studies

Health state	GBD 2015	GBD 2010	GBD 2004
Borderline intellectual functioning	0.011	0.0034	
Intellectual disability-mild	0.043	0.031	0.290
Intellectual disability-moderate	0.100	0.080	0.430
Intellectual disability-severe	0.160	0.126	0.820
Intellectual disability-profound	0.200	0.157	0.760

One possible explanation for the discrepancies is that health professionals and members of the general population used different definitions of “health” in making their judgments. Perhaps members of the general population consider an individual with ID to be disabled, but in otherwise excellent health because he or she does not have a “disease” as this term is understood by laymen. In contrast, health professionals might have interpreted the construct of health more broadly, taking into account not only physical health at a specific point in time, but also current and future well-being, including an individual’s ability to carry out activities of daily living and to fulfill his or her educational, occupational, and social potential. Such a broad interpretation of health is consistent with the definition of health articulated in the WHO constitution, “…a state of complete physical, mental and social well-being and not merely the absence of disease or infirmity.” From this perspective, the DWs assigned to the different levels of ID in the most recent GBD studies are surprisingly small, despite the fact that the lay descriptions clearly enumerate the limitations of individuals at each ID severity level (Table 2).

**Table 2:** Lay descriptions for different levels of severity of intellectual disability: GBD 2015

Severity levels	Lay descriptions
Borderline intellectual functioning	Is slow in learning at school. As an adult, the person has some difficulty doing complex or unfamiliar tasks but otherwise functions independently.
ID/mental retardation: mild	Has low intelligence and is slow in learning at school. As an adult, the person can live independently, but often needs help to raise children and can only work at simple supervised jobs
ID/mental retardation: moderate	Has low intelligence, and is slow in learning to speak and to do even simple tasks. As an adult, the person requires a lot of support to live independently and raise children. The person can only work at the simplest supervised jobs.
ID/mental retardation: severe	Has very low intelligence and cannot speak more than a few words, needs constant supervision and help with most daily activities, and can do only the simplest tasks.
ID/mental retardation: profound	Has very low intelligence, has almost no language, and does not understand even the most basic requests or instructions. The person requires constant supervision and help for all activities

The descriptions do not specify the IQ ranges associated with each category, but in the ICD10, mild ID corresponds to an IQ between 50 and 69; moderate ID to an IQ between 35 and 49; severe ID to an IQ between 20 and 34; and profound ID to an IQ below 20.

The view that the DWs for ID in GBD 2015 underestimate the diverse challenges of affected individuals, is reinforced by considering other health states with similar DWs. The DW of borderline intellectual functioning (0.011) is comparable those of amputation of a thumb (0.011), ear pain (0.013), abdominopelvic problem-mild (0.011), dental caries: symptomatic (0.010), and generic uncomplicated disease: anxiety about diagnosis (0.012). Health states with a DW similar to ID/mental retardation:moderate (0.100) include diabetic neuropathy (0.133), epididymo-orchitis (0.128), neck pain-moderate (0.114), infectious disease: acute episode-severe (0.133), and amputation of one arm: long-term with or without treatment (0.118). Even ID/mental retardation:profound (DW of 0.200), with a lay description indicating that an individual is unable to function independently in any capacity, has a DW lower than that of concussion (0.214) and fracture of the pelvis-short term (0.279).

As noted earlier, the duration of a disease contributes to the YLD, so a health state with a DW similar to that of one of the categories of ID but which is easily treated, with full recovery and only rare complications (e.g., epididymo-orchitis), will produce many fewer DALYs than an essentially untreatable, life-long condition such as ID. On the other hand, if a thumb were amputated in childhood, its YLD would be similar to the YLD of borderline intellectual functioning, despite the fact that the two individuals would face distinctly different challenges over their lifespans.

In order to fully capture the burden imposed on an individual by the sequelae of exposure to a developmental neurotoxicant, the disease model applied must include not only the individual’s physical health at a given point-in-time, but also the extent to which exposure limits the likelihood that the individual will be able to reach his or her educational, occupational, and social potential in subsequent years. The need to consider such a developmental perspective, extending over the life course, is one way in which the health effects of a developmental neurotoxicant differ from those of many other risk factors considered in GBD studies. Although a reduction in IQ is the endpoint most often measured in developmental neurotoxicity studies, and has been considered in GBD studies, it is really only the “tip of the iceberg,” serving as a proxy for the myriad other neurodevelopmental impairments that are sequelae. Moreover, rather than being the end of the story for an affected child, a reduction in IQ might be only the beginning, and a failure to consider how life unfolds over time for the child with an impaired IQ will result in a gross underestimate of the magnitude of the impact that developmental neurotoxicant exposure has had, given that early-life exposures to neurotoxicants might initiate developmental cascades that, over time, manifest in other adverse health states. Although the lay descriptions of the different severity levels of ID in GBD 2015 refer to the limitations that these states place on an adult’s functioning, the respondents providing the judgments on which DWs are based did not appear to apply a life-course perspective. A recent longitudinal study in New Zealand provides a good example of the importance of such a perspective. In that study, a child’s blood lead concentration at age 11 years was inversely related not only to IQ in adulthood, at 38 years of age, but also to socioeconomic status (SES) [10]. Greater lead exposure in childhood was associated with reduced upward social mobility, and the SES of an individual with higher a blood lead concentration in childhood tended to be lower than that of his or her parents. The link reported between early lead exposure and anti-social behavior in adulthood provides another example of a developmental cascade, initiated by reduced IQ and mediated by a variety of downstream sequelae, including impairment of impulse control, inability to delay gratification, lack of educational achievement, ADHD, and substance abuse.

While disability weights are available for health states other than ID that have been associated with developmental exposures to neurotoxicants (Table 3), to date these health states have not been included in GBD studies of such exposures. It is surprising that the DW for ADHD is about 4 times that of borderline intellectual functioning, even though effective treatments are available to reduce the impact of ADHD and only accommodations are available to mitigate the impact of borderline intellectual functioning. One challenge in applying GBD methods to developmental neurotoxicants is that because the constellations of sequela might not be recognized as disease states, DWs are not available for them. One such example is chronic metallic mercury vapor intoxication. Steckling and colleagues used expert elicitation and a systematic review to develop a description of this health state and generated a DW by asking expert respondents to complete a pairwise comparison exercise. The resulting DW was then used to estimate the global disease burden associated with mercury intoxication caused by artisanal gold mining [11].

**Table 3:** Disability Weights for Health States Relevant to Developmental Neurotoxicants

Health state	GBD 2015	GBD 2010	GBD 2004
ADHD	0.045	0.049	0.20
Conduct disorder	0.241	0.236	0.150
Asperger’s syndrome	0.104	0.110	
Autism	0.262	0.259	0.550
Hearing loss-mild	0.010	0.005	0.040
Speech problems	0.051	0.054	
Motor impairment-mild	0.010	0.012	0.010
Motor plus cognitive impairment-mild	0.031	0.054	0.024

In a GBD study, only risk factor-outcome associations for which the evidence regarding causality is considered convincing or probable should be included. Currently, the World Cancer Research Fund grades of evidence are used to identify associations that meet this criterion [1]. In this framework, greatest weight is given to evidence from randomized controlled trials, non-randomized intervention studies, and prospective observational studies. In studying developmental neurotoxicants, or environmental health risks in general, it is generally not feasible to conduct either randomized trials or interventional studies of risk-outcome associations. Therefore, the absence of evidence from such studies should not be weighted heavily in assessing the likelihood that such associations reflect causality. Other systematic review strategies that include consideration of evidence from non-human and toxicologic studies, as well as from human studies, would be more appropriate [12].

## Conclusion

Current GBD methods will not fully characterize the impacts of developmental neurotoxicants on population health. It would be unfortunate if priorities were set or policy decisions made based on how developmental neurotoxicants compare to other risk factors on the basis of DALYs calculated using these methods. While the results generated by applying these methods would be valid, they would capture only a small fraction of the total health impact of such exposures. In effect, the GBD method endorses “a high-risk” approach to disease prevention rather than a “population-based” approach. In an approach from the latter perspective, subclinical impacts would be considered to represent a health loss because of the toll they exact on an individual’s current and future well-being. Because exposure to some developmental neurotoxicants is ubiquitous, very large numbers of individuals suffer such impacts, resulting in a large cumulative toll on the well-being of a population. In addition, a burden analysis that is based solely on reduction in IQ scores ignores the contributions of the other health states that might occur in the future as a result of a cascade of adversities initiated by childhood exposure to a neurotoxicant. A second concern about the application of GBD methods to developmental neurotoxicants is the underestimation of the magnitude of the disability associated with health states that are the most common sequelae of these exposures. The explanation is not certain, but it might reflect a greater weighting of physical illnesses than limitations that prevent an individual from achieving as much success as an adult as he or she might otherwise have achieved. An approach based on a human capital framework, which both encompasses, “the knowledge, skills, competencies, and attributes embodied in individuals that facilitate the creation of personal, social and economic well-being,” ([13], p.18), and acknowledges the “dynamic complementarities” implicit in the concept of developmental cascades holds greater promise of fully estimating the disease burden attributable to neurotoxicants. It is not clear that current GBD methods can accommodate such an approach. One of the important advantages of a GBD analysis is that it permits direct comparison of the health burdens associated with diverse risk factors or diseases. Developing methods that remedy the problems of GBD methods as they pertain to developmental neurotoxicants without applying them to other risk factors and diseases would eliminate this advantage, so careful thought needs to be given to how to retain the ability to conduct comparative assessments but, at the same time, provide estimates of the disease burden that are more policy-relevant.

## Abbreviations

ADHD: Attention Deficit Hyperactivity Disorder
DALY: Disability-Adjusted Life-Year
DW: Disability weight
GBD: Global Burden of Disease
ICD: International Classification of Disease
ID: Intellectual disability
IQ: Intelligence Quotient
NHANES: National Health and Nutrition Examination Survey
OECD: Organisation of Economic Co-operation and Development
WHO: World Health Organization
YLD: Years lived with disability
